# MRTF: Basic Biology and Role in Kidney Disease

**DOI:** 10.3390/ijms22116040

**Published:** 2021-06-03

**Authors:** Maria Zena Miranda, Zsuzsanna Lichner, Katalin Szászi, András Kapus

**Affiliations:** 1 Keenan Research Centre for Biomedical Science of the St. Michael’s Hospital, Toronto, ON M5B 1W8, Canada; zena.miranda@mail.utoronto.ca (M.Z.M.); zsuzsanna.lichner@unityhealth.to (Z.L.); katalin.szaszi@unityhealth.to (K.S.); 2 Department of Surgery, University of Toronto, Toronto, ON M5T 1P5, Canada; 3 Department of Biochemistry, University of Toronto, Toronto, ON M5S 1A8, Canada

**Keywords:** actin cytoskeleton, Rho GTPases, transcription factors, nucleocytoplasmic shuttling, gene expression, profibrotic epithelial phenotype, myofibroblast, kidney fibrosis

## Abstract

A lesser known but crucially important downstream effect of Rho family GTPases is the regulation of gene expression. This major role is mediated via the cytoskeleton, the organization of which dictates the nucleocytoplasmic shuttling of a set of transcription factors. Central among these is myocardin-related transcription factor (MRTF), which upon actin polymerization translocates to the nucleus and binds to its cognate partner, serum response factor (SRF). The MRTF/SRF complex then drives a large cohort of genes involved in cytoskeleton remodeling, contractility, extracellular matrix organization and many other processes. Accordingly, MRTF, activated by a variety of mechanical and chemical stimuli, affects a plethora of functions with physiological and pathological relevance. These include cell motility, development, metabolism and thus metastasis formation, inflammatory responses and—predominantly-organ fibrosis. The aim of this review is twofold: to provide an up-to-date summary about the basic biology and regulation of this versatile transcriptional coactivator; and to highlight its principal involvement in the pathobiology of kidney disease. Acting through both direct transcriptional and epigenetic mechanisms, MRTF plays a key (yet not fully appreciated) role in the induction of a profibrotic epithelial phenotype (PEP) as well as in fibroblast-myofibroblast transition, prime pathomechanisms in chronic kidney disease and renal fibrosis.

## 1. Introduction

Rho family GTPases are prime regulators of the cytoskeleton [1]). Precise cytoskeletal control, in turn, is a prerequisite for normal renal structure and function. For example, the delicate morphology of podocyte foot processes, the structural basis of the filtration barrier, is fundamentally dependent upon subtle and highly regulated F-actin organization [2,3,4,5]. The cytoskeleton is also the structural basis of cell motility. It is thus not surprising that alterations in the activities of Rho family proteins, either due to genetic defects or provoked by cellular injuries, are associated with, and play significant pathogenic roles in acute and chronic kidney diseases [6,7,8,9]. However, the cytoskeleton affects organ function not only through its structural roles; the cytoskeleton is a regulator of gene expression as well. It exerts this (somewhat less appreciated yet not less important) function predominantly by controlling the nucleocytoplasmic shuttling of a select set of transcription factors. This mechanism renders the cytoskeleton a cell fate-determining device, i.e., a key determinant of phenotype transitions, plasticity, cell survival/death and injury/repair, critical processes in health and disease. Based on these functions, the cytoskeleton emerges as an essential “structure-function converter”, a signaling hub that integrates an array of diverse chemical (e.g., cytokines) and mechanical (e.g., pressure, tissue stiffness) inputs and links them to gene expression [10,11,12]. Rho GTPases, responsive to both mechanical and chemical stimuli [13,14,15], represent a major afferent arm of this transcriptional regulation. Central among the Rho family/cytoskeleton-controlled transcriptional regulators (coactivators) is the myocardin-related transcription factor (MRTF) family (see Section 2, Section 3 and Section 4), comprising of two isoforms, MRTF-A and B, encoded by separate genes. In this review we use the term “MRTF” when the effect of the two isoforms is not specifically distinguished. Substantial evidence has been accumulating that MRTF, and its nucleus-resident transcription factor partner, serum response factor (SRF) play important roles in proliferation, phenotype control (e.g., epithelial-mesenchymal transition, fibroblast-myofibroblast transition), secretory profile, matrix generation, oxidative state, intermediate metabolism, cytoskeleton organization, contractility and cell migration, and thereby in the pathogenesis of multiple disease states (see Section 5). MRTF, primarily through its transcriptional and epigenetic actions, has emerged as a crucial mediator of organ fibrosis [16,17,18]. Considering the kidney, MRTF has been implicated in fibrogenesis in the context of diabetic and obstructive nephropathy as well as in the pathobiology of acute kidney injury (see Section 6). Indeed, MRTF contributes to a slew of distinct fibrogenic processes in various renal cell types, including podocytes, tubular, mesangial and endothelial cells, fibroblasts and macrophages, encompassing the glomerular, tubular, interstitial and immune compartments (Section 5 and Section 6). Ubiquitously expressed in both epithelial and mesenchymal cells, MRTF is also a key factor in the crosstalk between these tissue compartments (epithelial-mesenchymal interaction), a process of key importance during injury/repair. Finally, it is worth noting that MRTF forms interactive networks with other major fibrogenic transcriptional regulators, including the TGFβ-controlled Smad proteins [19,20], and the Hippo pathway effectors, YAP and TAZ [21,22,23], all of which are involved in the pathogenesis of (renal) fibrosis. The nuclear accumulation of YAP and TAZ are also directly regulated by the acto-myosin cytoskeleton [24], representing another major pathway whereby Rho family GTPases affect gene expression. 

In this overview, we will summarize pertinent information regarding the basic biology of MRTF and its role in renal pathobiology, highlighting the underlying cellular and molecular mechanisms. We point to outstanding questions and hope to raise awareness about the central importance of this transcriptional regulator in kidney disease, and about its potential exploit as a therapeutic target.

## 2. MRTFs: Their Discovery and Modus Operandi

SRF was long known to activate two disparate programs: it can drive immediate early genes (like c-fos), involved in growth factor-mediated cell proliferation, and it can also induce expression of muscle genes, specifying a tissue differentiation program [25,26,27,28,29] (reviewed in [30]). However, the molecular mechanism that could act as a switch between such distinct functions remained an enigma for a while. It was then discovered that (1) RhoA-activating mediators, such as lysophosphatidic acid, can stimulate SRF-dependent transcription, and (2) the active forms of RhoA, Rac1 and Cdc42 are also potent inducers of this process [31,32]. Moreover, while SRF was known to associate with members of the ternary complex factor (TCF) family [33], and the TCF/SRF complex was shown to bind to gene promoters via neighboring cis-elements (TCE, and SRE, respectively), Rho family-induced activation of SRE was TCF-independent [31,34,35]. Importantly, actin polymerization per se was shown to drive SRF-dependent transcription [35,36]. These results forecasted the existence of another SRF interactor that might link this transcriptional pathway to Rho GTPases and the cytoskeleton. The breakthrough arrived with the near-parallel discovery of MRTF (also known as Megakarocytic Acute Leukemia (MAL); megakaryoblastic leukemia (MKL) or Basic, SAP And Coiled-Coil Domain (BSAC) Protein) and its mode of action by the Olson [37] and Treisman laboratories [38]. The identification of MRTF and its modus operandi explained both the Rho-dependence of the SRF pathway (see below), and the disparate roles of SRF as an early gene inducer vs. a differentiation driver. The former action manifests when SRF is in complex with TCF, and the latter when SRF pairs up with MRTF or myocardin, the eponymous, (cardiac) muscle-specific family member [39].

Initially myocardin was identified in a search for muscle-specific SRF activators [40]. It is expressed in smooth and cardiac muscle during development but is restricted to cardiac muscle in adult life. Myocardin binds to and activates SRF, which occupies the CC(A/T)rich_6_GG cis element or GArG box (the canonical SRE) on the DNA. Myocardin belongs to the SAP (SAF-A/B, Acinus, PIAS) family of chromatin-remodeling proteins, and similar to other members, it is constitutively nuclear. Importantly it is not activated by Rho GTPases. From here, the identification of MRTFs and their connection to Rho signaling followed two (converging) paths. Based on gene homology, Wang et al., from the Olson group cloned *mouse MRTF-A* and *MRTF-B* [37], and showed that these proteins are expressed in a large variety of tissues and organs, and are potent activators of SRF-dependent transcription. It was also uncovered that MRTF-A is identical with MAL, a previously cloned *human* protein, the gene of which undergoes chromosomal translocation (t(1;22)) and fusion with the gene encoding OTT, an RNA-binding protein [41,42]. The resulting gene product, OTT-MAL, is a deregulated, constitutive activator of SRF [43,44] and the pathogenic culprit of megakaryoblastic acute leukemia [42,44]. Finally, coming from an immunological angle, BSAC, an anti-apoptotic transcription factor was described, which exhibited strong capacity to activate CArG-containing promoters [45], and proved to be identical with MRTF.

**Figure 1:**
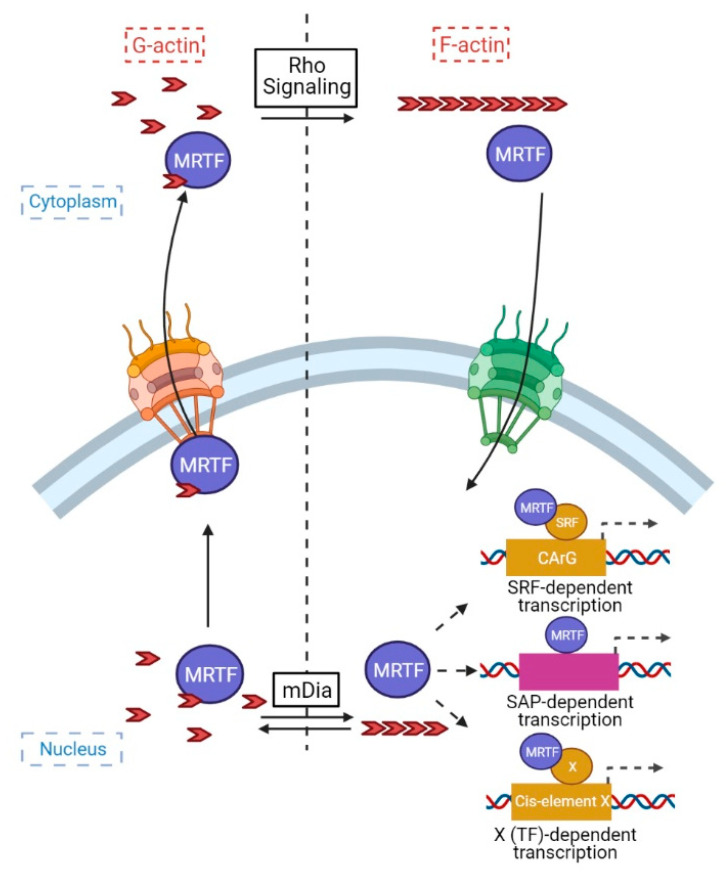
The regulation of MRTF subcellular localization and transcriptional activity by the actin cytoskeleton MRTF-A/B are transcriptional coactivators regulated by F/G actin ratio. Cytosolic G-actin binds MRTF masking a Nuclear Localization Sequence (NLS) (see Figure 2). Activation of small Rho GTPases promotes F-actin polymerization, thereby reducing the cytosolic G-actin pool, and causing G-actin to dissociate from MRTF to unmask the NLS, which enables importin α/β-dependent nuclear translocation. In the nucleus (1) MRTF binds SRF forming the MRTF/SRF complex which induces the transcription of various genes (SRF-dependent transcription); (2) MRTF can independently facilitate transcription of genes via its SAP domain (SAP-dependent transcription); and (3) MRTF can bind other TFs (e.g., smad3, TAZ) which induce transcription via alternative non-CArG dependent cis-elements. Additionally, the nuclear F/G actin ratio regulates MRTF function. Increased mDia activity enhances MRTF/SRF transcriptional activity, while nuclear G-actin binding to MRTF supports CMR1-dependent nuclear export. Together, the actin cytoskeleton acts as an integral regulator of MRTF-dependent gene expression (dotted arrows). (Created with BioRender.com, accessed on 15 April 2021).

In parallel with these genetic studies, Miralles et al., in the Treisman lab [38] identified MAL (MRTF) as the RhoA/actin-regulated SRF activator, thereby finding the hitherto missing link. As opposed to myocardin, MRTF is predominantly cytosolic in resting cells and Rho activation induces its nuclear translocation (Figure 1).

Moreover, the effect of Rho is conferred by actin remodeling. MRTF is an actin monomer- (G-actin) binding protein and its association with G-actin inhibits its nuclear accumulation. This is, in part, due to G-actin-mediated masking of MRTF’s nuclear localization signal (NLS) [67] but also to the faster export of the MRTF/G-actin complex from the nucleus [68,69]. These findings unraveled a strikingly elegant and powerful mechanism. MRTF rapidly cycles across the nuclear membrane in resting cells. Upon cytosolic Rho activation, due to the ensuing F-actin assembly, G-actin “is stolen” (dissociates) from MRTF, facilitating its import; in parallel nuclear Rho activation, via the ensuing nuclear actin polymerization, inhibits MRTF efflux. Furthermore, actin binding to MRTF in the nucleus also inhibits its transcriptional activity [12,68]. Taken together, compartmentally regulated increases in actin polymerization shift MRTF to the nucleus, and unleash its transcriptional potential. A plethora of factors (e.g., guanine nucleotide exchange factors (GEFs), GTPase activating proteins (GAPs) [9,70,71,72] regulate Rho GTPases and/or induce posttranslational modification of proteins involved in actin sequestering (e.g., thymosin [73]), severing (e.g., cofilin [36,74,75,76]) and polymerization (e.g., mDIA [69,77]) or modify actin itself [78]. All of these regulate MRTF, which thus emerged as a major highway connecting cytoskeletal state to gene expression.

## 3. Structure

**Figure 2:**
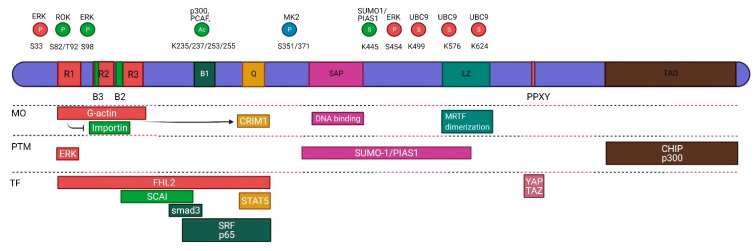
Schematic of MRTF domain architecture, co-factor interaction, and posttranslational modification. MRTFs contain 3 N-term RPEL domains (R1–3) enabling actin binding, 2 interspersed basic amino acid regions (B3, B2) containing the nuclear localization sequence (NLS); Leucine Zipper (LZ) which is necessary for MRTF dimerization and MRTF/SRF-dependent transcription; B1 region, Q region, SAP domain, PPxY motif, and a Transcriptional Activation Domain (TAD), all of which are necessary for binding various regulators and co-factors to regulate MRTF-dependent transcription. Posttranslational medications (PTM) of these domains regulate MRTF nuclear accumulation and/or transcriptional activity (see Table 1). Residues indicated include those known to be modified by phosphorylation (P), acetylation (Ac) or SUMOylation (S), with the modifiers noted above. Green indicates that the residue/PTM enhances MRTF activity, red indicates that the residue/PTM suppresses MRTF activity, and blue indicates no known role. Molecules with known interaction sites within MRTF are depicted spanning the domains which they are suggested to bind. This includes: (1) regulators of MRTF activity (Figure 1); (2) regulators of PTM with known binding sites; and (3) transcription factors (TF) or co-factors, which bind to MRTF and regulate downstream gene activation (see Table 2). (Created with BioRender.com, accessed on 01 June 2021).

MRTF-A (MAL, MKL1) and MRTF-B (MAL16, MKL2) are encoded by different genes (chromosomes 22 and 16 in *human*, respectively) but share a high level of homology and a similar domain structure. They contain three N-terminal RPEL motifs (named for the corresponding amino acids), interspersed with two basic regions (B3, B2) comprising a bipartite NLS, followed by another basic region (B1), a glutamine-rich sequence (Q), the family-defining SAP domain, a coiled-coil leucine zipper (LZ) motif and a C-terminal transactivation domain (TAD) (Figure 2).

The RPEL motifs are the G-actin binding sites, which along with two spacer regions allow the formation of a pentameric G-actin/MRTF complex [79,80]. Occupancy of the N-terminus by 5 actins buries the bipartite NLS, thereby inhibiting importin α/β-dependent nuclear import [80,81,82]; in fact, the importin α/β complex competes with actin for the RPEL motif [81]. Association of actin with the spacer regions (between the RPELs) may also be necessary for exportin-1- (CRM-1)-mediated export [79,83]. Moreover, actin binding is cooperative, and the trimeric G-actin/MRTF complex is still mainly cytosolic. Thus, MRTF could respond sharply to changes in G-actin concentration around a threshold level [79]. Overexpression of non-polymerizable G-actin [38], or actin depolymerization by the monomer sequestering drug, latrunculin A completely abolishes nuclear MRTF accumulation, as expected. However, paradoxically, Cytochalasin D or Swinholide, two toxins that also induce F-actin disassembly, are potent inducers of MRTF nuclear translocation because they disrupt the MRTF-G-actin interaction [36,84].

The B1 basic domain is also necessary for nuclear import [38], and—together with the Q-rich stretch—for the binding of SRF [85]. The Q-domain also harbors one of the leucine-rich sequences (L2), which serves as a CRM1-dependent nuclear export signal (NES). L1 is located in the RPEL region, and disruption of either L1 or L2 induces nuclear accumulation [83]. The SAP domains function as adaptors in protein-protein interactions and have DNA binding capacities, through which they regulate chromatin organization [86,87]. Accordingly, MRTF can also directly contact DNA [85], a feature that has at least two important functional connotations: first, MRTF (partly through this mechanism) may affect transcription in an SRF-independent manner, and second, MRTF emerges as an important epigenetic factor [63,88,89,90,91] regulating the histone code (acetylation, methylation) and chromatin remodeling. These actions represent an important facet of the pathophysiologic roles of MRTF, as will be detailed in the context of various disease states. The LZ motif is involved in homo- and heterodimerization of MRTF isoforms [39,92], which may impact the rate of MRTF shuttling and thus duration of its action, constituting an understudied aspect of MRTF regulation. The TAD lends potent transcriptional activity to MRTF-interacting transcription factors, primarily to SRF but also alternative partners (e.g., SP1 and Smads, see below). 

## 4. Regulation

MRTF activity is controlled primarily by its nuclear import and export, which are in turn dictated by the state of the actin cytoskeleton, set by a large cohort of Rho family regulators and effectors, as discussed above. Furthermore, cytoskeleton-regulating proteins may affect MRTF not only through their direct effect on cytoskeleton dynamics (actin polymerization). For example, actin nucleation-promoting factors N-WASP, WAVE2 and JMY activate MRTF because they directly compete with it for G-actin binding through their WH2 domain [93,94]. In addition to the level of available G-actin, MRTF is also regulated by two other main factors: its posttranslational modification and interaction with other regulatory proteins. These inputs impact MRTF’s localization, transcriptional activity and stability.

### 4.1. Posttranslational Modifications

It was observed early on that activation of either the Ras or the Rho signaling pathway elicits upward shifts in the molecular mass of MRTF, consistent with phosphorylation [38]. Indeed, MRTF contains 26 putative S/T phosphorylation sites, which can positively or negatively modify its activity [46,47] (see Table 1). Based on pharmacological and/or biochemical approaches, a variety of kinases have been implicated in MRTF phosphorylation including ERK [38,47], p38 MAPK (p38) [95] and its downstream effector MK2 [48], as well as GSK3β [49]. These can either directly or indirectly alter MRTF phosphorylation and consequently its localization or stability. Rho kinase (ROK), the most plausible link connecting Rho signaling to MRTF phosphorylation also impacts MRTF localization and activity [16,96] but it remains unclear if this involves direct phosphorylation. Some pertinent mechanisms are summarized below.

ERK-mediated phosphorylation of S98 in the RPEL motif of MRTF-A facilitates nuclear accumulation by inhibiting actin binding [46]. This finding suggests that in addition to the cellular G-actin level, G-actin affinity of MRTF can also be modulated, which calibrates its cytoskeletal sensitivity without a change in the cytoskeleton per se. Moreover, the same kinase can target multiple sites, with differing effects: e.g., ERK-mediated phosphorylation of S454 in the LZ motif was reported to promote nuclear exit of MRTF, presumably because it facilitates G-actin binding that supports nuclear efflux [47]. Thus, ERK may induce increased nuclear import (“on” effect) followed by enhanced nuclear export (“off” effect), overall accelerating MRTF shuttling.

The activation of p38, downstream from cell-cell contact disruption [97], osmotic stress [76] or TGFβ stimulation [95] is required for nuclear accumulation and/or increased transcriptional activity of the already nuclear MRTF. We found that pharmacological inhibition or siRNA-mediated downregulation of p38 reduced the TGFβ-induced shift in the molecular mass of MRTF, indicating an effect of phosphorylation [95]. Inhibition of p38 also prevented the nuclear accumulation of MRTF in vivo in a right ventricular pressure overload-induced cardiac fibrosis model [98]. Relevantly, p38 has a paramount role in organ (e.g., renal) fibrosis [99], and the p38 inhibitor Pirfenidone—the sole antifibrotic drug in clinical practice [100,101] was also shown to reduce TGFβ-induced MRTF phosphorylation in tubular cells [95]. Nonetheless the scenario is complex as SRF is also phosphorylated and activated by p38 [102]. Finally, the p38 effector MK2 was shown to directly phosphorylate MRTF (S351, S371) but the functional consequences remain unclear [48]. Taken together, p38 has emerged as an important and therapeutically targetable modulator of MRTF, but future work is warranted to address the exact underlying mechanisms.

GSK3β was described to phosphorylate myocardin and suppress its transcriptional activity, thereby limiting atrial natriuretic factor-induced hypertrophy of cardiomyocytes [103]. Subsequently, we have shown that GSK3β is recruited to MRTF by Smad3, and induces phosphorylation, consequent ubiquitination and degradation of MRTF in tubular cells. β-catenin competes for Smad3 with GSK3β, and thus it stabilizes MRTF [49]. Since GSK3β is the key enzyme responsible for β-catenin degradation as well, and both β-catenin and MRTF are important mediators in organ (e.g., kidney) fibrosis, GSK3β could suppress fibrogenesis, in part, by promoting the degradation of these transcriptional regulators. Phosphorylation-dependent ubiquitination of myocardin is mediated by E3 ligase C terminus of Hsc70-interacting protein (CHIP) [50]. While proteasomal degradation of MRTF has been well documented [49,104], the phosphodegron and the responsible ubiquitin ligases remain to be identified.

**Figure 3:**
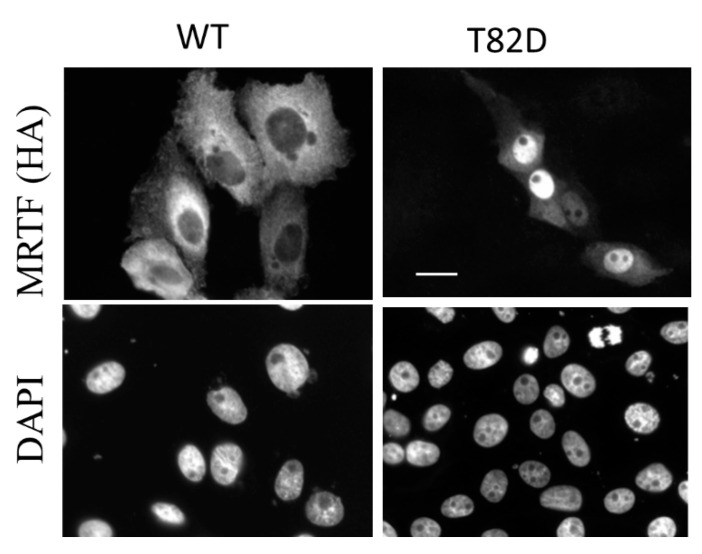
Phosphomimetic mutation of a potential ROK target site induces nuclear localization of MRTF. LLC-K1 proximal tubular cells were transfected with Hemagglutinin (HA)-tagged WT or T82D mutant *MRTF-B* constructs. Cells were stained for the HA epitope and nuclear marker, DAPI. Note that WT MRTF is cytosolic whereas the T82D mutant shows robust nuclear accumulation. Bar: 20 µm. (P. Speight and & A. Kapus, unpublished).

As mentioned above, active Rho provokes MRTF phosphorylation [38], and ROK inhibition abrogates S1P- [96,105], TGFβ- [106], osmotic stress- [76] or force transmission-induced [74] MRTF activity. However, to our best knowledge, direct phosphorylation of MRTF by ROK has not been shown (as yet). Nonetheless MRTF does contain ROK target motifs, and we found that phosphomimetic mutations of these (S82D and T92D) in the MRTF-B RPEL domain redistributes MRTF from the cytosol to the nucleus in tubular cells (P. Speight and A. Kapus, unpublished observation, (Figure 3). Thus, it is conceivable that ROK directly targets MRTF, which in turn might impact its actin affinity, a hypothesis worthwhile of further study.

Such putative effect may be superimposed on the indirect-cytoskeletal—impact of ROK on MRTF, which seems to be brought about by two mechanisms. First, ROK promotes actin polymerization by activating LIM kinase, which phosphorylates and thereby inhibits cofilin, leading to reduced actin severing and higher F/G actin ratio [107]. Indeed, the operation of this pathway was shown to be critical for the serum induced rise in SRF activity [108] and for the force-triggered nuclear translocation of MRTF [74]. Second, ROK promotes myosin light chain phosphorylation and thereby myosin-dependent contractility [109]. Intriguingly, inhibiting myosin activity by a dominant negative myosin light chain mutant or by the drug blebbistatin dramatically reduced TGFβ- or cell contact injury-induced, MRTF-dependent activation of the α- smooth muscle actin promoter, and contact disassembly-induced MRTF translocation in kidney tubular cells [16]. These findings suggest that contractility per se might be a regulator of MRTF. While the underlying mechanisms remain to be defined, acto-myosin dependent force transmission from the extracellular matrix and the cytoskeleton to the nucleus via the linker of the nucleoskeleton to the cytoskeleton (LINC) complex [110] emerges as a significant regulator of the traffic of mechanosensitive transcription factors, including MRTF [111,112,113]. The LINC complex and the nucleoskeleton may act by modulating extra- or intranuclear actin assembly [111,112] or the permeability of the nuclear pore complexes [114,115]. Interestingly both the LINC complex and myosin were shown to regulate genome-wide transcriptional responses, with a subset of common target genes [116]. Future work should explore the role of the LINC complex and myosin in the control of MRTF-dependent transcription, a potentially important facet of mechanotransduction and the pathobiology of mechanical stress-related fibrogenic states (tissue stiffness and distension). This aspect may have particular relevance in renal pathologies since myosin mutations (e.g., in *MYH9*) show strong association with chronic kidney disease [117]. Considered together, phosphorylation plays a major role in MRTF regulation, and the combinatorics of 26 target residues provides enormous versatility for fine-tuning. Much remains to be learned about this type of control under physiological and pathological conditions.

In addition to phosphorylation, MRTF is controlled by acetylation (Table 1). The histone acetyl transferase (HAT) p300 was shown to acetylate myocardin [51], and it likely mediates MRTF acetylation as well, as HAT and MRTF can form a complex [52]. p300-mediated acetylation was associated with increased MRTF/SRF activity on promoters of muscle genes (e.g., *MYH9, MYL9* as well as those encoding the matricellular protein Cyr61 [52]). Conversely, MRTF interacts with Histone Deacetylase 6 (HDAC6) [56], an enzyme localized predominantly in the cytosol, HDAC5 [54] (present in both cytosol and nucleus), and Sirtuin 1 (SIRT1) [57], a NAD-dependent deacetylase, localized in the nucleus. While each can deacetylate MRTF, the functional consequences were different. HDAC6 inhibition (i.e., increased acetylation) promoted MRTF activity to drive SRF-dependent α-smooth muscle actin expression; similarly HDAC5 overexpression (reduced acetylation) mitigated SRF/MRTF-mediated induction of antiapoptotic genes in neuronal cells [54]. In keeping with this, HDAC5 inhibition (increased acetylation) promoted the MRTF-dependent proinflammatory gene transcription by stimulating the activity of the MRTF/Nuclear Factor Kappa B (NFκB) (p65) complex in macrophages [55]. In contrast, SIRT1 overexpression (i.e., reduced acetylation) increased the capacity of MRTF to activate the collagen I promoter [57]. Together these results raise two important points. First, acetylation is a powerful modulator of MRTF, which may have a role in the promoter-selective regulation of its activity. The overall impact of acetylation may depend on a variety of factors including (a) the given promoter region; (b) TFs that MRTF partners with on a promoter; (c) the particular lysine residues targeted by acetylation/deacetylation; and (d) the cellular compartment in which MRTF is deacetylated. Second, MRTF forms complexes with histone-modifying enzymes at promoters, wherein its own modification by these may be a regulator of MRTF’s epigenetic and transcriptional effects.

Sumoylation of myocardin was reported to increase its activity on cardiogenic genes [59], while—interestingly—MRTF sumoylation at three lysine residues were shown to have a suppressive effect. Rho enhanced MRTF sumoylation [58], which may act as an inbuilt break in the control of MRTF activity. Again, this aspect of regulation warrants further studies.

### 4.2. Regulatory Protein Interactions

In addition to posttranslational modifications, MRTF is controlled by physical interactions with a number of proteins (Table 2). Four and half LIM domain protein 2 (FHL2), a multifunctional adaptor, is both a target (an MRTF/SRF-dependent gene) and a regulator of SRF/MRTF signaling. It binds to both SRF and MRTF, and while it stabilizes MRTF, it can either inhibit or stimulate the transcriptional activity of the SRF/MRTF complex, depending on the MRTF isoform and the particular promoter [60,61]. Interestingly FHL2 has been shown to be upregulated in and contribute to the pathogenesis of a variety of renal diseases, including interstitial fibrosis in diabetic, obstructive or hypertensive nephropathy [118,119,120,121,122]. Importantly, fibroblast-specific deletion of FHL2 attenuated myofibroblast accumulation (α-SMA expression) and matrix deposition (collagen, fibronectin) in the unilateral ureteral obstruction (UUO) model of renal fibrosis [122]. The likely mechanism is that FHL2 is a potent activator of the Wnt/β-catenin and TGFβ/β-catenin pathways (major drivers of renal fibrosis), and accordingly its absence abrogates the nuclear accumulation and activity of β-catenin [118,122]. FLH2 also binds to focal adhesion kinase (FAK), which in turn activates Rac; this pathway was proposed to underlie podocyte effacement in hypertensive kidney disease. Indeed, FLH2 knockout mice were partially protected against hypertension-induced albuminuria [121]. Since renal fibrogenesis is associated with MRTF overexpression [123,124], and MRTF is a driver of FHL2 gene, it is conceivable that MRTF plays a key role in increased FHL2 expression associated with these pathologies. Moreover, positive feedback loops may augment these effects, since both β-catenin and FHL2 can stabilize MRTF, contributing to its upregulation. 

Suppressor of cancer cell invasion (SCAI) is a binding partner and inhibitor of MRTF, which reduces cancer cell migration primarily by suppressing the MRTF-induced β1 integrin production in cancer cells [125]. Remarkably, SCAI is downregulated during clinical and experimental renal fibrosis [126], which may be one of the reasons for enhanced MRTF signaling in these pathologies, as will be addressed further in the corresponding sections. Finally, a variety of transcription factors can bind to MRTF, including Smad3, TAZ/YAP, SP1, NFκB. These interactions impact not only (mutual) transcriptional activities but also the nuclear traffic of MRTF; the interplay between MRTF and these TFs will be further discussed in the relevant sections.

### 4.3. Regulation of MRTF Transcription 

MRTF is also regulated at the transcriptional level, although the underlying mechanisms remain largely uncharacterized. Nonetheless, β-catenin has been recently identified as an inducer of the MRTF gene [127], suggesting that β-catenin not only stabilizes MRTF but also enhances its synthesis. While this observation was made in a tumor context, it may well be relevant in organ fibrosis as well. A very recent report shows that YAP can also activate the transcription of MRTF [128]. Since MRTF is a key transcriptional regulator of TAZ [22,95,124], MRTF and the hippo pathway effectors TAZ/YAP appear to form a positive feedback loop in each other’s transcriptional control. Future studies should address other regulators of MRTF expression. It is worth noting that several microRNAs have been implicated in MRTF expression (e.g., [129,130]). This branch of research is in a nascent state but will likely unearth key mechanisms in regulation of MRTF expression.

## 5. Targets, Functions, Actions

MRTF regulates a whole spectrum of processes, including development (of skeletal, cardiac, smooth muscle, neuronal and hematopoietic tissues), cell migration, phenotype shifts (e.g., EMT), mechanotransduction, wound healing/regeneration, extracellular matrix formation, cell cycle control, lipid and glucose metabolism and others. Accordingly, MRTF is involved in several pathologies, most prominently in cancer metastasis formation and organ fibrosis (for reviews of the various aspects see [11,17,18,125,131,132,133,134,135,136,137,138,139]). Overall, these widespread functions are brought about by three sets of mechanisms: MRTF can act as a

(1) transcriptional co-activator for SRF or other TFs
(2) regulator of epigenetic processes and chromosome organization
(3) “moonlighting” protein, when it exerts its effect independent of its role in gene expression.

Each of these mechanisms plays important roles in physiological and pathological processes.

### 5.1. MRTF as a Transcriptional Coactivator

The most complete assembly of MRTF/SRF target genes was published by Esnault et al. [140], using MRTF chromatin-immunoprecipitation followed by deep mRNA sequencing (ChIP-seq) in fibroblasts. To identify genes likely regulated by MRTF, stringent criteria were used including (a) serum-inducibility; (b) MRTF ChIP; (c) inhibition by latrunculin A; c) stimulation by Cytochalasin D. Of the serum-inducible set, 921 genes showed at least one other criterion, and 683 genes exhibited all of them, with 398 genes containing a CArG box within 2 kB of the transcription start site (“direct”) and 285 genes within 70 Kb (“near”). Gene ontology (GO) analysis revealed that the corresponding gene products are related to cytoskeleton/focal adhesions, development, transcription, signaling, growth and metabolism, in accordance with the results of functional studies. Here we provide examples of “famous” target genes that were shown to be (a) MRTF-dependent in kidney cells and/or (b) directly related to renal pathologies. These include the contractility protein myosin [16,141], the myofibroblast hallmark α-SMA [16,20,104,142,143], actin-severing (cofilin), capping (CapZ) [20]) and bundling (filamin) [144]) proteins; focal adhesion and cell-matrix components (α and β integrins [145]), tenascin [144,146]); extracellular matrix proteins (collagen [62,147], CTGF [124,148,149,150]); caveolar proteins (caveolin 1 [144,151]), fibrogenic cytokines (TGFβ [124]), cell cycle regulators (p21 [152]), NADPH oxidases [153,154]; and transcription factors, encompassing SRF itself [20], the EMT drivers Snai1/2 and ZEB2 [155,156,157] and the Hippo effector TAZ [22,95]. Moreover, SRF/MRTF-targeted CArG motifs are frequently located in the vicinity of AP1 and TEAD sites (that are activated by YAP/TAZ), showing that these TFs often regulate the same genes, and may act synergistically with MRTF [23]. In addition to driving protein genes, MRTF was shown to induce microRNAs as well in CArG-dependent manner [158]. An intriguing example is miR-21, which is responsible for the “mechanical memory” of mesenchymal stem cells [159]. When plated on stiff surfaces these cells mobilize a fibrogenic program, and through MRTF-induced upregulation of miR-21 they “remember their stiff past” (for as long as two weeks) even when subsequently placed on soft surfaces. This mechanism is of major significance since fibrogenic phenotype shifts represent a serious impediment in stem cell therapies.

MRTF can impact gene expression not only by pairing up with the “canonical” SRF, but also with other TFs. These “alternative” interactions can also contribute to (renal) pathogenesis. In these cases MRTF does not act via the CArG box but through the cis-element targeted by its alternative partner. E.g. in kidney tubular cells TGFβ was shown to induce the interaction of MRTF with Smad3 [19,20] and this complex can drive, through a non-conventional Smad-binding element, the transcription of Snai2 (a.k.a SLUG), a key EMT-provoking gene suppressor. Snai2 in turn induces the downregulation of epithelial junction proteins (e.g., E-cadherin) [19]. Interestingly, while Smad3 is necessary for this CArG-independent MRTF action, binding of Smad3 to MRTF inhibits, the “classic” CArG-dependent effect of MRTF, e.g., the induction of the α-*SMA* promoter [20]. Thus, Smad3 has the capacity to act as a switch, redirecting MRTF to alternative targets (see also next section). The collagen promoter contains a non-canonical CArG box adjacent to a specificity protein-1 (SP1) sites. Interestingly, mutating these sites or the SP1 inhibitor mithramycin abrogated the MRTF-induced activation of the collagen promoter. This indicates that SP1 is necessary for the action of MRTF and the two factors work synergistically [62]. MRTF also drives the TNFα-induced expression of the RhoA/Rac GEF, GEF-H1 in tubular cells in an SP1-dependent manner (S. Venugopal, A. Kapus and K. Szaszi, manuscript in preparation). MRTF can also interact with Stat5, and this complex appears to be critical for driving fibronectin and ICAM-1 expression-through Stat5 sites—in mesangial cells stimulated by advanced glycation end product (AGEs) [65], which accumulate during diabetes and aging [65]. Further, MRTF was shown to physically interact with the NF-κB subunit p65 (RelA), resulting in a mutual inhibition of the action of these TFs. This mechanism underlies the bone morphogenic protein 4 (BMP-4)-induced anti-inflammatory response in vascular smooth muscle cells [64], and the reduction in ICAM-1 expression in endothelial cells [160]. On the other hand, MRTF was reported to promote lipopolysaccharide (LPS)-provoked, p65-dependent induction of inducible nitric oxide synthase (iNOS). Binding of MRTF to p65 modifies the histone mark at the *iNOS* promoter [63,91]. These studies indicate that CArG-independent actions of MRTF are also important regulators of gene expression, and they might be inductive or suppressive, depending on the particular genes or the cellular context. Furthermore, ChIP-seq analysis revealed that MRTF can act as a gene suppressor as well. This understudied area is a fertile ground for future research.

### 5.2. MRTF as an Epigenetic Modifier

Epigenetic control (e.g., via histone methylation, acetylation and chromatin remodeling) is a major factor in the regulation of gene expression, and MRTF impacts this process by several mechanisms (Table 3). MRTF deficiency erased key histone modifications in LPS-stimulated macrophages. As a determinant of the “trimethyl landscape”, MRTF recruits SET1, the H3K4 trimethyl transferase to NFκB sites in the promoters of proinflammatory genes [161], thereby stimulating their expression. H3K4 methylation is also enhanced via the interaction of MRTF with Ash2/Wdr5 methylation complex in response to angiotensin induction of the endothelin gene [162]. Interestingly MRTF was also reported to be important for demethylation of certain loci (H3K9) [163,164] via recruiting demethylases (e.g., KDM3A); these can also augment expression of select set of genes, e.g., those for NADPH oxidases [164]. MRTF regulates histone acetylation (e.g., at H3K9, H3K18, H3K27, H4K16) as well by recruiting various histone acetyl-transferase components, including p300, pCAF, TIP60 and MOF [52,91,123,164]. Conversely, through interaction with histone deacetylases (as detailed in Section 6) MRTF could contribute to acetylation/deacetylation cycles. Finally, MRTF controls chromatin remodeling too: e.g., it can recruit Brg1/Brm, components of the SWI/SNF ATP-dependent chromatin-remodeling complex to endothelin [165] and other promoters [162]. Since many of these modifications are relevant in various models of kidney disease, an integrated functional view will be provided in Section 6.

### 5.3. ”Moonlighting” Functions of MRTF

Our recent studies (manuscript under revision) indicate that both SRF and MRTF are necessary for serum-induced resorption of the primary cilium (PC). The PC is a microtubule-based, membrane-surrounded mechanochemical antenna [166,167] that acts as a flow sensor in the tubular epithelium [168,169], and a key regulator of cell division, differentiation and metabolism in all nucleated cells [170,171,172]. Ciliary defects are associated with a large set of severe diseases, so called ciliopathies, the prime examples of which are various forms of polycystic kidney disease (PKD) [173,174,175,176]. The PC is a dynamic organelle that develops from the basal body (BB) that arises from the membrane-anchored mother centriole as the cell leaves the cell cycle, and gets resorbed during cell cycle reentry. PC dissolution is necessary for cell proliferation because it liberates the BB, which in turn can function as a mitosis organizing center [177,178,179,180]. We found that MRTF downregulation eliminates serum-induced PC resorption and facilitates ciliogenesis. While SRF and MRTF can impact the cell cycle and may also drive genes necessary for cilium resorption, our results suggest that their role in PC regulation goes beyond such transcriptional effects. Remarkably, they localize to the PC or the BB, and MRTF physically interacts with cilium resorption proteins (e.g., Aurora A kinase), and ciliogenesis regulators (e.g., CEP290). In accordance with these findings, a recent BioID screen by the Gingras group showed that MRTF can be in complex with various BB/PC/centrosomal proteins (https://prohits-web.lunenfeld.ca (accessed on 15 April 2021). While the mechanistic details await further elucidation, these findings point to the fact that MRTF and SRF operate not only as TFs. Such moonlighting functions signify a new chapter in MRTF biology. In addition, they raise the possibility that MRTF might play a role in the pathogenesis of cystic diseases.

### 5.4. MRTF Knockouts

Given the wide range of regulated genes and processes, it is surprising that MRTF-A knockout mice are grossly normal. However, they are unable to nurse their offspring, because MRTF-A is indispensable for the proper development and function of mammary myoepithelial cells, and thus for milk ejection [181,182].

The likely reason for the lack of other defects is that MRTF-B (or myocardin) may fulfill most of MRTF-A’s critical functions. Nonetheless, MRTF-A KO animals show altered responses in a wide range of disease models and are useful tools to define the pathobiologic roles of MRTF (detailed for the kidney in Section 6). In contrast, MRTF-B knockouts are embryonically lethal due to serious cardiovascular defects related to the failure of differentiation of smooth muscle cells in the brachial arteries [183]. Thus, deletion of both *MRTF-A* and *B* can be achieved only in a tissue-specific manner. This has been accomplished in the context of the kidney, namely in podocytes, the highly specialized epithelial cells of the glomeruli. This cell type is a very relevant choice, as tight control of its subtle actin skeleton is indispensable for proper foot process formation, dynamics and slit diaphragm structure, which in turn are indispensable for normal glomerular filtration. A recent elegant study indicates that podocyte-specific deletion of either *SRF* or *MRTF-A* and *B* (but not one of them alone) causes foot process effacement, proteinuria, azotemia and reduced expression of podocyte markers [184]. These findings indicate that the SRF/MRTF system plays a fundamental physiologic role in the kidney. Currently no tubule-specific MRTF-A and/or B knockout animals exist; their generation would greatly facilitate the assessment of the physiologic and pathologic functions of MRTF in that compartment.

## 6. MRTF in Kidney Diseases

Considering the widespread role of MRTF in many processes, it is important to provide an overall conceptual framework to interpret its contribution to kidney disease. The major unifying notion is the role of MRTF in phenotype shifts, predominantly as it relates to organ fibrosis. To highlight these common mechanisms, first we will summarize those (mostly in vitro) studies that allowed insight into the cellular basis of this function. 

### 6.1. The Epithelium as a Key Initiator of Fibrosis

Fibrosis is a dysregulated form of regeneration characterized by excessive ECM deposition, and the consequent disruption of tissue architecture and function [185,186]. Regarding the kidney, essentially all chronic renal diseases, from congenital disorders (like PKD or Alport syndrome) to acquired nephropathies (e.g., of diabetic, hypertensive, immune-mediated or obstructive origin) culminate in kidney fibrosis, which histologically manifests as glomerulosclerosis and/or tubulointerstitial fibrosis [187,188]. The cellular culprit of fibrosis is the myofibroblast, a contractile and ECM-producing cell type hallmarked by the expression of α-SMA. Myofibroblasts can originate from a multitude of cell types (fibroblasts, endothelial and epithelial cells, pericytes, bone-marrow derived fibrocytes), and the identification of their sources has been the focus of intensive research for over two decades (reviewed in [135,189,190,191,192]. It was recognized early on that epithelial (tubular) injury (e.g., due to high glucose levels, hypoxia, hydrostatic pressure, etc.) is one of the key initiating events of kidney fibrosis. Moreover, epithelial injury was also shown to be sufficient to trigger robust renal fibrosis. For example, proximal tubule-specific (Six2 Cre-driven) expression of diphtheria toxin (DT) receptor allows selective injury of this compartment upon administration of low dose DT. This proximal tubule-targeted insult resulted in robust tubulointerstitial fibrosis (TIF) and glomerulosclerosis [193]. Further support for the prime role of the epithelium came from genome-wide association studies (GWAS), characterizing single nucleotide polymorphism (SNPs) in chronic kidney disease (CKD). These studies revealed that approx. 20% of CKD cases can be linked to certain single gene variants, while others have a polygenic background with an overall heritability of 30–50% [194,195,196,197,198]. However, only 5% of these are localized in coding regions of the genes, pointing to the role of dysregulated expression. Recent elegant work aimed at linking a given SNP to the actual disease-causing gene built on the hypothesis that diseases are cell-type-specific, and therefore genetic variants are localized to cell type-specific regulatory regions [199]. To address this presumption, the authors used laser capture microdissection to isolate the glomerular and the tubular compartments from 151 kidneys, and then performed a compartment-specific “expression quantitative trait loci (eQTL)” analysis. Integrating the previous GWAS data, single cell RNA sequencing and epigenetic studies, they defined correlations between the observed SNPs (eVariants) and the potential corresponding target genes (eGenes) in a compartment-specific manner. This analysis identified 27 genes which showed differential expression according to the presence of the particular GWAS variants. Importantly, large portion of these occurred in (or were restricted to) the proximal tubules. They concluded that “renal proximal tubules show the greatest enrichment for GWAS–eQTL target genes (39%)”.

### 6.2. EMT/EMyT and PEP

**Figure 4:**
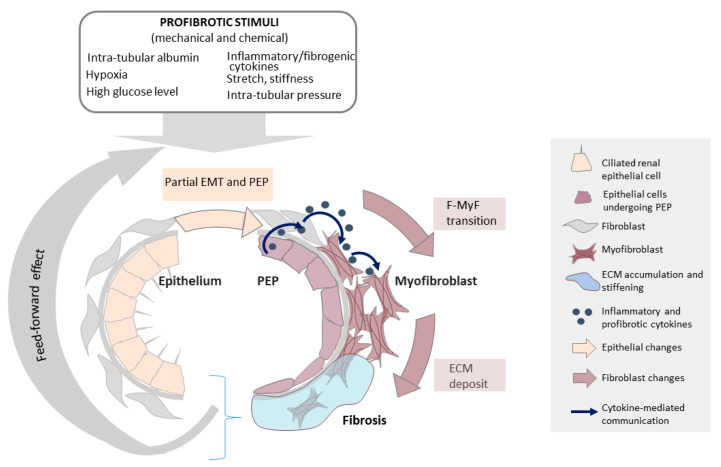
Profibrotic epithelial phenotype (PEP) drives fibrosis. Epithelial injury can be secondary to mechanical forces (stretch and stiffness, intratubular pressure) or chemical insults (high glucose level, hypoxia), that result in the loss of cell-cell contacts, and inflammation. Together, these events drive a partial epithelial-to-mesenchymal transition, and the acquisition of the consequent profibrotic epithelial phenotype (PEP), characterized by increased production and secretion of profibrotic cytokines. PEP cells communicate with the adjacent stroma via the released cytokines (epithelial-mesenchymal crosstalk), that stimulate fibroblast-to-myofibroblast transition (F-MyF). Myofibroblasts secrete additional cytokines that trigger their proliferation, while they also deposit ECM components, leading to stiffening. On the other hand, PEP reinforces profibrotic stimuli, creating a feed-forward loop of injury, reinforcement of PEP and accelerated fibrosis.

While the epithelium initiates the fibrotic process, mesenchymal cells (fibroblasts, myofibroblasts) are its executors. How is then epithelial initiation linked to mesenchymal execution? One potential explanation that dominated the field for a long time was that the epithelium is a key source of (myo)fibroblasts through epithelial-mesenchymal (EMT) or epithelial-myofibroblast (EMyT) transition (Figure 4). This idea proved to be a fertile ground to get insight into the underlying cellular pathobiology.

Since the hallmark of the myofibroblast is α-SMA expression, the regulation of this process in epithelial cells emerged as a key question. The fact that renal epithelial cells can, in vitro, transform to fibroblasts and myofibroblasts have been documented by a plentitude of studies [142,200,201]. Moreover, the α-*SMA* gene, harboring two CArG boxes in its promoter is a typical SRF/MRTF target. Accordingly, MRTF is regulated by fibrogenic stimuli [16,97] and is indispensable for epithelial α-SMA expression [97,104]. These early studies allowed us to link MRTF to fibrosis for the first time [16,97]. The critical inducer of this transformation is TGFβ, the main fibrogenic cytokine. However, TGFβ- while necessary-is not sufficient for tubular EMT/EMyT. Remarkably the intact epithelium is resistant to such transformation, and a second hit is needed, which can be supplied by either the disruption/uncoupling/absence of the intercellular contacts (which can be induced by wounding, subconfluence or low extracellular calcium concentration) [16,20,97,202,203,204] or by mechanical stress (induced by restricted cell geometry, matrix stiffness, etc.) [22,205,206,207]. The common factor underlying this second hit is increased cellular tension or contractility, a result of small GTPase activation and cytoskeleton remodeling. All of these are potent regulators of MRTF. Interestingly, TGFβ alone is a poor inducer of MRTF nuclear translocation [20,95,104,208], while contact injury/mechanical stress triggers robust rise in nuclear MRTF levels but this is a transient phenomenon. However, TGFβ augments and prolongs the nuclear accumulation of MRTF [20]. The α-*SMA* promoter, which contains cis-elements for three major fibrogenic TF systems—SRF-MRTF (CArG boxes), Smad3 (SBEs) and Yap/TAZ-TEAD (TBEs) side-by-side proved to be an invaluable model to define the role of these TF pathways under various fibrogenic conditions. Investigation of the mechanism underlying the synergy between the arms (contact injury and TGFβ) of the two-hit model of α-SMA expression gave surprising results. TGFβ and injury indeed drive the α-*SMA* promoter synergistically but the SBEs are dispensable for this effect; synergy only requires intact CArG boxes, suggesting that in this system TGFβ acts primarily by potentiating the effect of MRTF. Moreover, these studies also allowed the definition of distinct phases of the transition. The first is an early Smad3-phase, when MRTF and Smad3 collaborate to induce a partial EMT. This results in the loss of some epithelial characteristics and enhanced mesenchymal gene expression. As mentioned above the Smad3/MRTF complex can induce the expression Snai1/2, major EMT-provoking TFs [19]. The second is a late Non-Smad3 phase, when MRTF alone turns on a myogenic program [20]. In contrast to the first phase, the second or myogenic phase is inhibited by Smad3, a finding consistent with reports showing that Smad3 levels decrease with the progression of (renal) fibrosis [209]. Later studies also revealed that combined humoral (TGFβ) and mechanical (cyclic stretch) stimulation reorders the relationship among the critical TFs. Namely, combined stimulation favors the association of Smad3 with TAZ. This has two consequences: first, it liberates MRTF from the MRTF/Smad3 and MRTF/TAZ complexes. Second it allows Smad3 and TAZ to reach their own cis-elements in gene promoters (e.g., α-*SMA*) [95].

While early fate-tracing studies proposed that the tubular epithelium is a major contributor to the myofibroblast population [210,211], more advanced lineage tracing methods refuted this scenario (reviewed in [135,190,212]. EMyT is a rare event in situ and only a small fraction (1–5%) of myofibroblasts is thought to be generated from the tubular epithelium in various animal models of fibrosis. Instead of a full EMyT, the epithelium undergoes a partial EMT, characterized by expression of EMT-promoting TFs (Snai1 and Twist), the loss of solute transporters, upregulation of mesenchymal markers, cell cycle arrest in G2, reduced fatty acid metabolism, and increased expression of fibrogenic mediators (TGFβ1) [156,213] and reviewed in [191] and [214]. Although partial, this mesenchymal shift is critical for fibrogenesis. Accordingly, epithelial deletion of Snai1 or Twist mitigated fibrosis in various animal models (UUO, folic acid, nephrotoxic serum), while epithelial overexpression in transgenic animals induced fibrogenesis [156,213]. These studies indicate that partial EMT is not only a feature but also a driver of renal fibrosis. How can MRTF be integrated into this refined paradigm? Two important points must be made. First, MRTF, a master regulator of EMT is a significant contributor to partial EMT as well. In fact, instead of “partial EMT” we introduced the more accurate term of “profibrotic epithelial phenotype” (PEP) [124], since the process is not restricted to the features of EMT (Figure 4). It also involves oxidative reprogramming exemplified by the increased expression of NADPH oxidases (e.g., Nox4 [153]), metabolic reprogramming, and most importantly a shift toward a secretory phenotype [124,156,215,216,217,218,219]. The injured epithelium starts producing fibrogenic cytokines (e.g., TGFβ, CTGF, PDGF, Hedgehog ligands) and MRTF silencing or pharmacological inhibition (CCG-1423) suppresses the synthesis of these mediators as well as Nox4 expression [124,153]. The produced cytokines in turn act on fibroblasts, thereby forming the link between the epithelial and the mesenchymal compartment (epithelial-mesenchymal crosstalk). MRTF also induces the expression of TAZ [22,95], another major fibrogenic TF in the kidney [124,153,203,220,221], which also contributes to cytokine production [124]. Evidence for the MRTF dependence of these processes in vivo (animal models) will be discussed in the Section 6.3. Second, the role of MRTF is likely different in the epithelial and mesenchymal compartment. While in the epithelium MRTF contributes to fibrosis via PEP (Figure 4), in most cases the process does not go “all the way”, i.e., the epithelium only rarely transforms to myofibroblast. This inbuilt break against a myogenic program, which is also manifest in the TGFβ resistance of the epithelium, is worthy of further study. In contrast, in the mesenchymal compartment MRTF is a key contributor to fibroblast-myofibroblast transition and in fact it is the main driver of this myogenic transition [17,222,223]. Indeed, MRTF has been described as major transcriptional driver of fibrosis in a wide variety of organs (including heart, lung, liver, skin, eye) and disease models [95,123,124,128,153,224,225,226,227,228,229,230,231,232,233,234,235,236,237]. Taken together, on the cellular level MRTF contributes to fibrosis both by partial EMT/PEP and by driving fibroblast-myofibroblast transition (Figure 4). In addition, as will be detailed in Section 6.3.3 it also impacts immune cell regulation.

### 6.3. MRTF in Animal Models of Kidney Disease

#### 6.3.1. Diabetic Nephropathy (DN)

Diabetes is one of the leading causes of CKD, and conversely ≈30% of diabetic patients (over 460 million people worldwide) develops CKD, indicating the tremendous medical and social implications of this condition. The first direct indication that MRTF may play an important role in DN came from the studies of Fintha et al., who showed that the MRTF inhibitor protein SCAI is downregulated in the kidneys of diabetic rats concomitant with the development of fibrosis [126]. Definitive evidence was then obtained by Xu et al., who used two models of DN, high fat diet (HDF) and streptozotocin (STZ)-induced diabetes, and compared renal function and histology in WT and MRTF-A-deficient (KO) mice [123]. In both models, the absence of MRTF-A improved the serum albumin/creatinine ratio or mitigated urinary albumin excretion, reduced Type IV and Type I Collagen expression, immune cell (lymphocyte, neutrophil and macrophage) infiltration, mesangial matrix expansion and TIF. In addition, both HDF and STZ increased MRTF-A mRNA levels in WT mice. Importantly, ChIP revealed that both insults increased the binding of MRTF to the promoters of the collagen genes Col1a1 and Col1a2. To provide mechanistic insight the authors used WT or KO fibroblasts in vitro and showed that high glucose induced MRTF engagement of the collagen promoters. They then performed multiple ChIP assays using various antibodies against either particular histone marks or the enzymes involved in their generation. Overall, they found that high glucose is associated with histone acetylation (in histone 3 at lysine 18 and 27, H3K18Ac and H3K27Ac), and trimethylation (H3K4Me3). Absence or downregulation of MRTF prevented these modifications. Similar observations were made in DN mice as well. Finally, they found that high glucose triggered the recruitment of the histone acetyl transferase p300 and the histone trimethylase component WDR5 to collagen promoters, both of which were necessary for collagen promoter activation. Importantly, genetic deletion or silencing of MRTF prevented the recruitment of both enzymes. The same lab extended these studies to the induction of CTGF, a critically important fibrogenic mediator [90]. CTGF was well known to be driven by MRTF via a classic CArG box [143,238], but this recent report indicates the involvement of epigenetic mechanisms as well. CTGF induction is reduced in MRTF KO mice. In addition to the above-described changes in histone acetylation, STZ or high glucose (in vitro) decreased H3K9 dimethylation (H3K9Me2) at the *CTGF* promoter, and this effect was also prevented by the absence/downregulation of MRTF. These results were recapitulated in primary renal tubular epithelial cells as well, indicating the importance of MRTF in epithelial gene expression. Finally, the change in H3K9Me2 was attributed to the recruitment of the histone demethylase KDM3A, a process that required MRTF. KDM3A and MRTF formed a complex at the *CTGF* promoter. Taken together, MRTF expression and activity increase during DN, and MRTF significantly contributes to the ensuing pathology, at least in part, via epigenetic modulation of gene transcription. Whole body MRTF-A KO animals are substantially protected against the loss of kidney function and the development of fibrosis, which suggests that—somewhat unexpectedly—MRTF-B cannot substitute for MRTF-A in his regard. Compartment- and isoform-specific regulation remains to be elucidated. A concise summary of MRTF’s involvement and the underlying mechanisms in DN-associated and other kidney diseases is provided in Table 4.

#### 6.3.2. Obstructive Nephropathy (ON)

Obstructive uropathy is the most prevalent cause of CKD in children [247], and unilateral ureteric obstruction (UUO) is the most frequently used rodent model of TIF [248]. UUO is a model of mechanically induced fibrosis, inasmuch as the initiating factor is an increase in hydrostatic pressure along the nephron. The first indication of the potential involvement of MRTF came from studies showing that during UUO the expression of the NAPDH oxidase Nox4 increases in the tubular epithelium, and MRTF was shown to be necessary for the injury-induced induction of Nox4 in vitro in tubular cells [153]. In addition to MRTF, TAZ—a downstream target of MRTF—was also required for Nox4 expression, and pharmacological inhibition of TAZ eliminated Nox4 expression in the UUO-challenged epithelium. In kidney fibrosis Nox4 was proposed to play both a protective role (at low levels) and a pathogenic one (and high levels) (reviewed in [249]), implying that MRTF may be involved in these processes. Next, Wang et al. [239] proposed that MRTF might play a fibrogenic role in UUO in the fibroblast compartment. Their studies were aimed at explaining the mechanism whereby AMP-activated kinase-α1 (AMPKα1), a major sensor of cellular energy levels, contributes to renal fibrogenesis [250]. During UUO AMPKα1 levels are increased, whereas global or fibroblast-specific deletion of AMPKα1 reduces fibrosis [239,250]. The mechanism was investigated in rat kidney fibroblasts in vitro; pharmacological activation of AMPKα1 led to increased cofilin phosphorylation (i.e., less F-actin severing), thereby promoting net F-actin polymerization. Concomitantly, the AMPKα1 agonist also induced nuclear translocation of MRTF, which was proposed to be causative of myofibroblast transition. While this could be one of the MRTF-activating mechanisms, our recent studies revealed that early UUO leads to robust RhoA activation, predominantly in the tubular epithelium [124]. Accordingly, we found increased nuclear translocation of both MRTF-A and B in focal regions of the tubular epithelium, providing direct evidence for MRTF activation in UUO. Moreover, MRTF-A and MRTF-B were increased at the mRNA and protein levels as well, an effect detectable already 24 h post-injury. To assess the functional significance of MRTF signaling, mice were treated with the MRTF inhibitor CCG-1423. In control animals UUO significantly increased the mRNA (or protein) levels of fibrogenic cytokines, TGFβ, CTGF, PDGF, and Indian hedgehog, and of these, CCG-1423 significantly suppressed TGFβ and CTGF production, concomitant with mitigating ECM deposition and α-SMA expression. MRTF inhibition also suppressed the induction of TAZ in the tubular epithelium. Parallel in vitro experiments have shown that mechanical stress (cyclic stretch) increased the synthesis of all of the above-mentioned cytokines in tubular cells, and each was suppressed—to a various degree—by pharmacological inhibition or downregulation of MRTF or TAZ. To further substantiate the PEP concept in vivo, we isolated the tubular compartment using laser capture microdissection and have shown that UUO enhanced tubular TGFβ, CTGF and TAZ expression, and each of these were reduced by CCG-1423 [124]. TAZ emerges as a major effector of MRTF, as evidenced by the fact that TAZ is strongly induced in various fibrotic conditions and its inhibition mitigates renal fibrosis [220,240,241]. Finally, the MRTF inhibitor SCAI was also downregulated in UUO [126]. Taken together, these findings show that MRTF expression and activity increase in obstructive nephropathy. Several mechanisms (RhoA- and AMPKα1 mediated cytoskeleton remodeling and SCAI downregulation-induced desinhibition) contribute to this process. In the mesenchymal compartment MRTF directly facilitates myofibroblast transition, while in the tubular epithelium it promotes PEP, leading to oxidative changes, TAZ and cytokine expression. Thus, the Rho/MRTF/TAZ pathway emerges as a key mediator of fibrosis and represents a potential therapeutic target.

#### 6.3.3. Acute Kidney Injury (AKI)

The most common causes of AKI, characterized by a sudden decrease in renal function, include ischemic insults, sepsis and poisoning, wherein hypoxia, pathogen- or damage-associated molecular patterns, inflammatory cytokines and environmental toxins or drugs damage the kidney, and particularly the tubular epithelium [251,252,253]. Its incidence is high (estimated to be 2–20% in hospitalized patients) and its mortality remains around 20% [251]. Of recent interest, AKI develops in 10% of hospitalized Covid-19 patients [254]. Beyond the acute phase, AKI was found to be associated with an 8.8-fold risk for CKD [255]. Thus, AKI represents a common and major clinical challenge. So far only one study addressed the role of MRTF in AKI. The results point to a novel cellular mechanism. Liu et al., in the Xu lab [154] used two AKI models: ischemia/reperfusion (I/R) and lipopolysaccharide (LPS) injection. Both insults caused deterioration of kidney function (blood urea nitrogen, plasma creatinine and urinary albumin/creatinine ratio), increased Kidney Injury Molecule-1 expression, high ROS production and a concomitant rise in renal Nox1 and Nox4 levels. All of these changes were ameliorated in MRTF-A knockout animals or upon MRTF inhibition by CCG-1423 in WT mice. Nox4 was upregulated in tubular cells but even more prominently in macrophages, while no change was observed in podocytes. This prompted the authors to concentrate on macrophages, showing that hypoxia/reoxygenation (H/R) induces MRTF-dependent Nox1 and Nox4 upregulation in these cells. Most importantly, myeloid cell-specific deletion of MRTF (crossing *MRTF* floxed mice with the Lyz2-Cre animals) was sufficient to improve renal function and reduce injury. Mechanistically, H/R caused histone modification, particularly H4K16 acetylation in the *Nox* promoters. The absence or inhibition of MRTF prevented these changes, as well as the recruitment of Myst1, one of the acetyl transferases that preferentially targets H4K16. Finally, a pharmacological Myst1 inhibitor ameliorated renal function and histological changes provoked by I/R. While this elegant study provided firm evidence for the role of MRTF in the pathogenesis of AKI, several questions remain open and are worthy of further study. Interestingly the major NADPH oxidase in macrophages is Nox2, whose deletion does not seem to alter the course of AKI [256]. Also, the mechanisms whereby MRTF act might be species-dependent, as the *human* proximal *Nox4* promoter contains an SRF/MRTF-regulated GArG box [153], which is not present in the *mouse* promoter. The exact mechanism through which MRTF binds to promoters to recruit the epigenetic machinery also remains to be elucidated. Further, besides epigenetic regulation, MRTF might act by transactivating other TFs, such as SP1 and NFκB, both involved in Nox regulation. Indeed, NFκB was shown to bind MRTF in macrophages [257]. Accordingly, MRTF may have a broad (and largely uncharacterized) role in the regulation of inflammatory gene expression [91,258]. The role of MRTF in determining macrophage phenotype (proinflammatory vs. regenerative) also requires further study. Clearly, as Liu et al. [154] have emphasized, further studies should define “a more holistic role for MRTF-A in AKI” in particular, and in inflammation in general.

#### 6.3.4. Polycystic Kidney Disease (PKD)—Solidifying a Hypothesis

PKD is the most common inherited renal disease (≈1:1000) [259,260] and its most prevalent form, Autosomal Dominant PKD (ADPKD) is caused by mutations in one of two primary cilium-associated membrane proteins, polycystin 1 (PC1 or PKD1, Pkd1 gene product, 85%) and polycystin 2 (PC2 or PKD2, *Pkd2* gene product, 15%) [175,261]. These proteins together can form a mechanosensitive cation channel. PKD is characterized by progressive cyst formation due to enhanced and spatially distorted tubular cell proliferation [259,262]. Although a large number of mutations have been described in the two genes, ADPKD is consensually regarded as a loss-of-function disease [261]. Beside cyst formation, the major histologic feature of ADPKD is robust fibrosis [263,264]. As such, its pathobiology is a prime example of dysregulated epithelial-mesenchymal communication because the causative defect (impaired PKD1 or PKD2 function) affects predominantly tubular cells, while the fibrotic matrix is the product of mesenchymal cells. Although the underlying cell biology is complex and involves multiple pathways (e.g., cAMP, mTOR, β-catenin signaling) [259,265], there is increasing evidence that abnormal Rho signaling plays an important role in the pathogenesis of the disease. The relevant facts include the following. Gene profiling performed on cysts and minimally cystic/normal regions of five PKD1 patients indicated significant enrichment of transcripts associated with mitogen-mediated proliferation, cell cycle progression, EMT, hypoxia, aging and immune/inflammatory responses. When the identified genes were classified according to corresponding TF networks, the SRF pathway proved to be one of the strongest signatures [242]. Direct evidence of the involvement of Rho was obtained in a recent study, which investigated alterations in gene expression in a *mouse* model with inducible tubular deletion of *Pkd1*. Under these conditions YAP and TAZ signaling was strongly upregulated. Looking for a plausible mechanism, the authors investigated Rho signaling and found that Rho activity (and accordingly ROK activity and myosin phosphorylation) was increased in Pkd1-deficient cells and animals. The absence of Pkd1 resulted in the membrane translocation (activation) of the Rho-GEF LARG, suggesting that PKD1 exerts tonic inhibition on this Rho activator, a key control mechanism lost in PKD. Moreover, inhibiting ROK or LARG reduced YAP/TAZ-dependent gene expression and the cystic phenotype. The authors argued that the PKD1-inhibited LARG-RhoA-ROCK→YAP/TAZ pathway enhances the expression of the proto-oncogene c-Myc, which in turn provokes excessive tubular cell proliferation and cyst formation. A very recent study reported that the loss of PKD1 is also associated with loss of ArhGAP35, a negative regulator of Rho, from the centrosome. This is accompanied by higher RhoA activity, actin reorganization and shorter cilia. The ROK inhibitor hydroxyfasudyl reversed cyst expansion [244]. While these studies clearly suggest that Rho activation can contribute to the dysregulation of cell proliferation and thus cyst formation, we propose that it may also underlie the other major feature of the disease i.e., fibrogenesis, by promoting PEP. Moreover, MRTF may play a key role in these transitions, both because it activates the SRF-dependent genes identified above, and because it induces the expression of TAZ per se. Further rationale for this hypothesis came from earlier studies showing altered vascular responsiveness in Pkd2+/− heterozygote mice [245,246]. These animals exhibit exaggerated vasoconstriction upon phenylephrin stimulation [245], although their intracellular Ca level is lower than the WT [266]. Searching for the underlying mechanism, Du et al. [246] showed that vascular smooth muscle cells from Pkd2+/− animals expressed more αSMA and exhibited higher F/G-actin ratio under resting conditions. Intriguingly, phenylephrine induced 3-fold higher Rho activation and 5-fold higher nuclear MRTF accumulation in Pkd2+/− smooth muscle cells that in WT controls. Thus, the loss of even one Pkd2 allele is sufficient to potentiate Rho/MRTF signaling in vascular smooth muscle. Finally, our ongoing studies suggest that MRTF is both overexpressed and shows enhanced nuclear accumulation in PKD1- or 2-deficient tubular cells and in the tubular epithelium of Pkd2-/- mice (Mei D, Lichner Z, Pei Y and Kapus A). These observations give strong support to the notion that enhanced MRTF signaling may be an important pathogenic factor in PKD, which could drive both cystogenesis and fibrosis. Conversely, inhibition of MRTF may signify a potential therapy. These possibilities prompt further research.

#### 6.3.5. Other Renal Diseases

The above sections summarize the documented involvement of MRTF in kidney diseases. However, this list likely represents only a fraction of the relevant pathologies. RhoA has been documented to play a role in a wide range of kidney pathologies, and in principle dysregulation of MRTF may play a role in each of these because dysregulated RhoA signaling can disrupt gene expression primarily via MRTF. Important examples include congenital and acquired proteinuric glomerular diseases, podocyte and mesangial cell injuries [8,9,267]. Further, any pathology associated with altered cytoskeleton organization is a potential “myrtfopathy”, impacting MRTF-dependent transcription. Examples include various forms of focal segmental glomerulosclerosis [268], or nephropathies associated with the MYH9, the myosin II heavy chain [117,268]. The emerging role of MRTF in the regulation of the primary cilium, both as a transcription factor and a cilium constituent, is fertile ground for future work. It remains to be explored whether MRTF can be linked to certain ciliopathies. Finally, new studies should be conducted to document enhanced MRTF signaling in *human* histological samples for various renal diseases. Such analyses will not only be important for assessing the role of MRTF in these pathologies, but may link altered MRTF signaling to different phases of these processes, thereby informing diagnosis and potentially therapy.

## 7. Perspectives: MRTF in Therapy

Given the substantial evidence indicating the role of MRTF in kidney diseases, a critical outstanding question is whether MRTF can be exploited as a pharmacological target in these conditions. This approach would be advantageous because it targets a hub downstream of a slew of stimuli, while at the same time it is less broad, more feasible and more appropriate for chronic conditions than inhibition of Rho itself. CCG-1423, the prototypic small molecule MRTF inhibitor was discovered as a suppressor of Rho/MRTF/SRF pathway downstream of Rho [269]. Strangely, its exact mechanism of action is yet uncertain: it has been proposed to bind directly to the RPEL motif of MRTF [270], to MICAL-2, a monooxygenase regulating actin oxidation and polymerization [78], and to pirin, a redox-sensitive nuclear protein [271]. Irrespective of the exact mechanism, chemical modification of the parent compound led to a series of MRTF inhibitors (e.g., CCG-100602, CCG-203971, CCG-222740, CCG-232601) with increased potency, better pharmacokinetics, stability, bioavailability and less toxicity [237,272,273,274]. Recently a high throughput screen identified a new and very potent class of MRTF inhibitors (5-Aryl-1,3,4-oxadiazol-2-ylthioalkanoic acids) [275]. While understanding the exact mechanisms of action of these drugs is an important goal for current research, there is firm evidence that various MRTF antagonists can mitigate tissue fibrosis in several models (lung, skin, heart, kidney, ocular, peritoneal etc.) [123,124,225,227,232,237,271,274]. They also effectively block metastasis formation in tumor models [273,276]. However, regarding kidney pathologies only the parent compound, CCG-1423 was tested, and only in a few models (DN, AKI, ON) [123,124,154]. The newer MRTF inhibitors are promising antifibrotics that deserve more attention and further scrutiny as experimentally—and potentially clinically-useful drugs. Treating fibrosis is a large unmet need, and MRTF inhibitors may, at least in part, hold the answer to this enormous problem.

## Figures and Tables

**Table 1:** Posttranslational modifications/modifiers of MRTF: phosphorylation, acetylation, and SUMOylation.

Enzyme	Site Modified	Domain Modified	Binding Site	Effect on MRTF Localization/Stability	Effect on MRTF Transcriptional Activity	Reference
**Phosphorylation**						
ERK	S98	between RPEL 1 and 2	RPEL1	Promotes MRTF nuclear import/prevents nuclear	+	[46]
ERK	S33	N-term of RPEL1	N/D	Promotes MRTF nuclear export	N/D	[46]
ERK	S454	Between SAP and LZ	N/D	Promotes G-actin binding and MRTF export	N/D	[47]
P38	N/D	N/D	N/D		+	
MK2	S351/371	Between Q and SAP	N/D	N/D	No effect	[48]
ROK	S82/T92	RPEL 1			+	
GSK3β	N/D	N/D	Binds MRTF via Smad3-dependent mechanism	Promotes MRTF ubiquitin-mediated degradation	−	[49]
**Ubiquitination**						
**Ubiquitinase**						
CHIP	N/D	N/D	TAD	Promotes MRTF ubiquitin-mediated degradation	−	[50]
**Acetylation**						
**Histone Acetyl Transferase (HAT)**						
P300	K235/237/253/255	N-term	Binds myocardin C-term	N/D	−/+	[51]
	Lysines are conserved between myocardin and MRTF	B1	Binds MRTFA C-term (TAD)	N/D	+	[52]
pCAF	Lysines are conserved between myocardin and MRTF	B1	N/D	Promotes MRTF nuclear translocation	+	[53]
**Histone Deacetyl Transferase**						
HDAC5	N/D	N/D	Binds MRTF (domain undescribed)	Prevents MRTF-A nuclear translocation	−	[54,55]
HDAC6	N/D	N/D	Binds MRTF (domain undescribed)	Regulates MRTF-A total protein (supresses)	−	[56]
SIRT1	Lysines are conserved between myocardin and MRTF	B1	Binds MRTF (domain undescribed)	N/D	+	[57]
**SUMOylation**						
UBC9	K499, 576, and 624	C-term region (C-term LZ?)	N/D	No effect	−	[58]
SUMO-1/PIAS1	K445	C-term region (C-term LZ?)	385–586 aa (C-term after SAP?)	No effect	+	[59]

N/D, Not Determined; − or + notes if the molecule affects MRTF transcriptional activity negatively or positively.

**Table 2:** Transcription factors and transcriptional co-factors interacting with MRTF.

Transcription Factor	Domain Bound	Effect on MRTF Localization/Stability	Effect on MRTF Transcriptional Activity	Reference
SRF	B1/Q	N/D	+	[37]
FHL2	N-term (RPEL/B1-3/Q)	Increased myocardin protein levels	+	[60,61]
	N-term (RPEL/B1-3/Q)	Increased MRTF-A protein levels	+	
	B1/Q	Decreased MRTF-B nuclear localization	−	
YAP/TAZ	C-term (PPxY)	Decreased MRTF nuclear accumulation	−/+	[22]
Smad3	B1	Promotes MRTF degradation	non-CArg = +; CArG = −	[19,20]
SP1	N/D	N/D	+	[62]
NFκB/p65	B1/Q	N/D	−/+	[63,64]
Stat5	Q	N/D	non-CArG/ICAM-1 = +	[65]
Stat3	N/D	N/D	+	[66]

N/D, Not Determined; − or + notes if the molecule effects MRTF transcriptional activity negatively or positively.

**Table 3:** MRTF epigenetic modifiers.

Epigenetic Modifier	Modification Type	Gene	Effect on Gene Activity	Reference
**Methylation**				
SET1	H3K4 trimethyl transferase	Proinflammatory genes	+	[161]
Ash2/Wdr5	H3K4 trimethyl transferase	Endothelin, COL1A1/COL1A2	+	[123,162]
KDM3A	H3K9 demethylase	CTGF	+	[90]
Jmjd1a	H3K9 demethylase	SMC differentiation markers	−	[163]
**Acetylation**				
p300	H3K18/H3K27 acetyltransferase	COL1A1/COL1A2	+	[123]
TIP60	H4K16 acetyltransferase	iNOS	+	[91]
MOF	H4K16 acetyltransferase	NOX1/4	+	[164]

− or + notes if the epigenetic modifier effects gene activity negatively or positively.

**Table 4:** MRTF in kidney diseases.

Disease	Animal/Cell Model	Experimental Conditions	Suggested Mechanim	Reference
Diabetic nephropathy	REC cell model and WT rat	SCAI overexpression, rat UUO	SCAI → blocks MRTF-A → locks fibrosis	[126]
	Mrtf-a KO mice and fibroblasts	In vivo (STZ, high fat diet), in vitro (STZ, high glucose)	MRTF-A is necessary to recruit histone acetyl- transferase and methyl- transferase to collagen promoters and activate type I collagen transcription	[123]
	MRTF-A KO mice and	In vivo (STZ, high fat diet) In vitro (STZ, high glucose)	MRTF-A regulates histone acetylation and methylation on the CTGF promoter, partially through interacting with KDM3A	[90]
Obstructive nephropathy	WT mice, REC cell model	UUO, in vitro functional studies	Epithelial MRTF-A links cytoskeletal and organization to redox state, through NOX4	[153]
	AMPK1α KO conditional (fibroblast)	UUO	AMPK1α → cofilin →F-actin → nuclear MRTF-A	[239]
	WT mice	UUO, MRTF-A inhibitor (CCG1423)	RhoA → MRTF-A → TAZ → PEP → fibrogenesis	[124,220,240,241]
	WT mice	UUO+ SCAI inhibition	SCAI → blocks MRTF-A → blocks fibrosis	[126]
Acute kidney injury	Macrophage-specific MRTF-A KO mice	Ischemia-reperfusion lipopolysaccharide	MRTF-A → MYST1 → H4K16Ac at *NOX* → ROS	[154]
Polycystic kidney disease	PKD patients	Microarray comparison	MRTF-A/SRF transcription network is upregulated	[242]
	PKD1 KO in tubules		Loss of PKD → LARG → RhoA → YAP/TAZ → c-Myc → cystogenesis	[243]
	PKD1 patients PKD1 KO mice	ROK-inhibitor (hydroxyfasudyl) treatment	Loss of PKD → ArhGAP35 → RhoA/ROK	[244]
	Pkd2+/−vascular smooth muscle	phenylephrin stimulation	Loss of PKD → RhoA → F-actin → αSMA	[245,246]
	Pkd1 and Pkd2 KO mice	Expression and localization studies	Increased MRTF expression and nuclear localization	Kapus lab, unpublished data

## Data Availability

Data sharing not applicable. No new data were created or analyzed in this study. Data sharing is not applicable to this article.
